# Automatic annotation of protein motif function with Gene Ontology terms

**DOI:** 10.1186/1471-2105-5-122

**Published:** 2004-09-02

**Authors:** Xinghua Lu, Chengxiang Zhai, Vanathi Gopalakrishnan, Bruce G Buchanan

**Affiliations:** 1 Dept. of Biostatistics, Bioinformatics and Epidemiology, Medical University of South Carolina, 135 Cannon St. Suite 303, Charleston, SC 29425, USA; 2 Dept of Computer Science, University of Illinois at Urbana-Champaign, 1304 W. Springfield Avenue, Urbana, IL 61801 USA; 3 Center for Biomedical Informatics, University of Pittsburgh, 200 Lothrop Street, Suite 8084, Pittsburgh, PA 15213 USA

**Keywords:** Gene Ontology, data mining, supervised learning, protein motif, feature extraction, logistic regression

## Abstract

**Background:**

Conserved protein sequence motifs are short stretches of amino acid sequence patterns that potentially encode the function of proteins. Several sequence pattern searching algorithms and programs exist foridentifying candidate protein motifs at the whole genome level. However, amuch needed and importanttask is to determine the functions of the newly identified protein motifs. The Gene Ontology (GO) project is an endeavor to annotate the function of genes or protein sequences with terms from a dynamic, controlled vocabulary and these annotations serve well as a knowledge base.

**Results:**

This paperpresents methods to mine the GO knowledge base and use the association between the GO terms assigned to a sequence and the motifs matched by the same sequence as evidence for predicting the functions of novel protein motifs automatically. The task of assigning GO terms to protein motifsis viewed as both a binary classification and information retrieval problem, where PROSITE motifs are used as samples for mode training and functional prediction. The mutual information of a motif and aGO term association isfound to be a very useful feature. We take advantageof the known motifs to train a logistic regression classifier, which allows us to combine mutual information with other frequency-based features and obtain a probability of correctassociation. The trained logistic regression model has intuitively meaningful and logically plausible parameter values, and performs very well empirically according to our evaluation criteria.

**Conclusions:**

In this research, different methods for automatic annotation of protein motifs have been investigated. Empirical result demonstrated that the methods have a great potential for detecting and augmenting information about thefunctions of newly discovered candidate protein motifs.

## Background

With the completion of many genome sequencing projects and advances in the methods of automatic discovery of sequence patterns (see Brazma [1] and Brejova et al [2] for reviews), it is now possible to search or discover protein sequence motifs at the genome level. If one regards protein sequences as "sentences" of the biological language with amino acids as the alphabet, then protein motifs can be considered as words or phrases of that language and determining the function of a motif is equivalent to determining the sense of a word. Identifying biological sequence motifs has been a fundamental task of bioinformatics, which has led to the development of several motif (pattern) databases, such as PROSITE, BLOCKS, SMART and Pfam [3-6]. These databases are usually constructed by studying the set of protein sequences that are known to have certain functions and extracting the conserved sequence motifs that are believed to be responsible for their functions. However, the number of motifs that can be extracted in this way is quite limited, and it has been a major challenge to discover new motifs. With the advent of algorithms and programs that can automatically discover sequence motifs from any given set of sequences [1,2,7-9], it is possible to mine a large number of sequences to find novel motifs without necessarily knowing their functions and to compile a dictionary of biological language accordingly. An essential task involved in the compilation of such a dictionary is to determine the function (the meaning) of newly identified protein motifs.

Here, we report development of general methods that can be used to predict the function of protein motifs by mining the knowledge in the Gene Ontology. The Gene Ontology™ (GO) project [10] is a concerted effort by the bioinformatics community to develop a controlled vocabulary (GO terms) and to annotate biological sequences with the vocabulary. A biological sequence is described in three different aspects, namely, biological process, cellular component, and molecular function. The standardized annotation with a controlled vocabulary is the main advantage of Gene Ontology, which facilitates both communications among scientists and information management. Both the number of annotated sequences and the number of GO terms associated with individual sequences in the Gene Ontology database are increasing very rapidly. Moreover, natural language processing techniques are also being used to automatically annotate gene products with GO terms [11,12]. Thus, it can be foreseen that the annotations of protein sequences in the Gene Ontology database will become more and more detailed, and have a great potential to be used as an enriched knowledge base of proteins.

The basic approach for determining the function of a motif is to study all the sequences that contain the motif (pattern). Intuitively, if all the functional aspects of the sequences matching a motif are known, we should be able to learn which function is most likely encoded by the motif, based on the assumption that every protein function is encoded by an underlying motif. This means that we would need a knowledge base of protein sequences, in which the functions of a sequence are annotated as detailed as possible. In addition, we would also need prediction methods that can work on a given set of protein sequences and their functional descriptions to reliably attribute one of the functions to the motif that matches these sequences. To determine the function of any novel motif, we would first search the protein knowledge base to retrieve all the functional descriptions of the proteins containing the motif, and then use such prediction methods to decide which function is encoded by the motif. In this research, we use the Gene Ontology database as our protein knowledge base and explore statistical methods that can learn to automatically assign biological functions (in the form of GO terms) to a protein motif.

Our approach is based on the observation that the Gene Ontology database contains protein sequences and the GO terms associated with the sequences. In addition, the database also contains information of known protein motifs, e.g. the PROSITE patterns that match the sequences. Thus, the protein sequences in the database provide a sample of potential associations of GO term with motifs, among which some are correct (i.e., the GO term definition matches the functional description of the motif) and some are not. This provides us an opportunity to perform supervised learning to identify discriminative features and use these features to predict whether a new association is correct or not. Current Gene Ontology database is implemented with relational database system, which allows one to perform queries like "retrieve all GO terms associated with the sequences that matches a given motif" and *vice versa*. However, the database usually returns more than one GO terms that may or may not describe the function of the motif in the query. Thus, we need methods to disambiguate which GO term describe the function of the motif (assign a GO term to a motif) and determine how confident we are as the assignment is concerned. We use statistical approaches to learn from known examples and cast disambiguation task into a classification problem. Furthermore, the probability output by the classifier can be used to represent its confidence for the assignment.

Recently, Schug et al [13] published their result of automatically associating GO terms with protein domains from two motif databases – ProDom and CDD [14,15]. Their approach is to use protein domains to BLAST [16] search against GO database and assign the molecular functional GO term from the sequence matching the domains with most significant *p*-value. They found that, in the database they worked with, most sequences only had one functional GO term. Therefore, they could assign the GO term of a sequence to the motif that matched with highest score with fairly good accuracy. However, due to restrictive assumption that each sequence has only one GO term, their approach can not address the potential problem that a sequence matching a motif has multiple associated GO terms, which is common case now, and how to resolve such ambiguity.

## Results

### The data set

We use the May 2002 release of the Gene Ontology sequence database (available online [17]), which contains 37,331 protein sequences. For each sequence, a set of GO terms assigned to the sequence is identified, and a set of PROSITE patterns that match the same sequence is also retrieved. If both sets are nonempty, all the possible pattern-term combinations formed by the two sets are produced. Table 1 shows an example association of GO terms with PROSITE motifs. The protein MGI|MGI:97380 from the database is assigned seven GO terms and the sequence also matches two PROSITE patterns. Thus, as cross product of two sets, 14 distinct associations are produced. Note that the same pattern-term association may be observed multiple times within the database. A total of 4,135 GO terms, 1,282 PROSITE motifs, and 2,249 distinct PROSITE-GO associations have been obtained from this database.

Using the information stored in the Gene Ontology and PROSITE, we manually judged a set of 1,602 cases of distinct PROSITE-GO associations to determine whether the association is correct or not. The PROSITE-GO association set has been judged in two different ways. One way is to label an association as correct if and only if the definition of the GO term and the PROSITE motif match perfectly according to the annotator. Gene Ontology has the structure of a directed acyclic graph (DAG) to reflect the relations among the terms. Most terms (nodes in the graph) have parent, sibling and child terms to reflect the relation of "belonging to" or "subfamily". The second way of judging GO-PROSITE association is to label an association as correct if the GO term and the PROSITE motif are either exact match or the definitions of GO term and PROSITE motif are within one level difference in the tree, i.e., the definition of GO term and the PROSITE motif have either a parent-child relation or a sibling relation according to the GO structure. Thus we have two sets of labeled PROSITE-GO associations, the perfect match set and the relaxed match set (with neighbors). Both sets are further randomly divided into training (1128 distinct associations) and test (474 distinct associations) sets. Since the test sample size is fairly large, the variance of the prediction accuracy can be expected to be small. Thus we have not considered any alternative split of training and test sets.

### Measuring term-motif associations

Intuitively, we may think of the GO terms assigned to a protein as one description of the function of a protein in one language (human understandable) while the motifs contained in the protein sequence as another description of the same function in a different language (biological). We would like to discover the "translation rules" between these two languages. Looking at a large number of annotated sequences, we hope to find which terms tend to co-occur with a given motif pattern. Imagine that, if the sequences that match a motif are all assigned a term *T*, and none of the sequences that do not match the motif is assigned the term *T*, then it is very likely that the motif pattern is encoding the function described by term *T*. Of course, this is only an ideal situation; in reality, we may see that most of, but not all of the proteins matching a motif pattern would be assigned the same pattern, and also some proteins that do not match the motif may also have the same term. Thus, we want to have a quantitative measure of such correlation between GO terms and motif patterns.

A commonly used association measure is mutual information (M.I.), which measures the correlation between two discrete random variables *X *and *Y *[18]. It basically compares the observed joint distribution *p*(*X *= *x*, *Y *= *y*) with the expected joint distribution under the hypothesis that *X *and *Y *are independent, which is given by *p*(*X *= *x*)*p*(*Y *= *y*). A larger mutual information indicates a stronger association between *X *and *Y*, and *I*(*X;Y*) = 0 if and only if *X *and *Y *are independent.

For our purpose, we regard the assignment of a term *T *to a sequence and the matching of a sequence with a motif *M *as two binary random variables. The involved probabilities can then be empirically estimated based on the number of sequences matching motif *M *(*NM*), the number of sequences assigned term *T *(*NT*), the number of sequences both matching *M *and assigned *T *(*NT*-*M*), and the total number of sequences in the database. Table 2 shows the top five terms that have the highest mutual information with PROSITE motif PS00109, which is the specific active-site signature of protein tyrosine kinases, along with the related counts.

**Figure 1:**
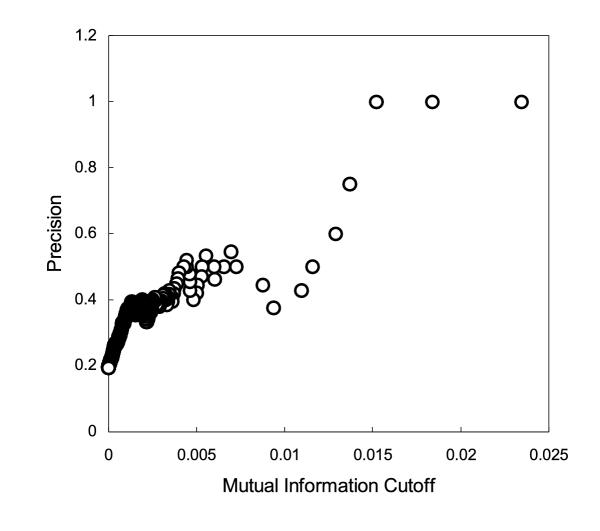
**Correlation of mutual information cutoff and term assignment precision**. Different M.I. cutoff value is used to assign GO terms to motifs. The precision of assignment is plotted vs M.I. cutoff value. The Pearson correlation coefficient between the precision and the cutoff is 0.837.

We set out to test whether we can use mutual information as a criterion to assign a GO term to a PROSITE motif. One approach is to use a mutual information cutoff value *c *to define a simple decision rule: assign term *T *to motif *M*, if and only if *I*(*T;M*) ≥ *c*. For a given cutoff *c*, the precision of term assignment is defined as the ratio of the number of correct assignments to that of the total assignments according to the cutoff *c*. In Figure 1, we plot the precision at different mutual information cutoff values. It is easy to see that, in general, using a higher (i.e., stricter) cutoff, the precision is higher; indeed, the Pearson correlation coefficient between the precision and the cutoff is 0.837. This suggests that mutual information is indeed a good indicator of the correlation

However, a drawback of such an approach is that, given a motif, sometimes, many observed motif-term associations can have mutual information above the cutoff value, making it difficult to decide which pair is correct. While in other cases, the mutual information of the observed motif-term pairs may all be below the cutoff value, but we still would like to predict what terms are *most *likely to be appropriate for the motif. To address this problem, we can use a different cutoff strategy, and adopt a decision rule that assigns a GO term to a motif based on the ranking of mutual information, which is a common technique used in information retrieval text categorization [19]. More specifically, for each PROSITE motif *M *in the annotated data set, all observed motif-term associations containing *M *are retrieved and ranked according to mutual information, then the term that has highest mutual information is assigned to *M*. Alternatively, if we use this approach to facilitate human annotation, we can relax the rule to include GO terms that have lower ranks, thus allowing multiple potential GO terms to be assigned to a motif, assuming that a human annotator would be able to further decide which is correct. In this method, the key in making a decision is to select a cutoff rank that covers as many correct associations as possible (high sensitivity) while also retrieves as fewer incorrect associations as possible (high specificity). The optimal cutoff can be determined by the desired utility function.

**Figure 2:**
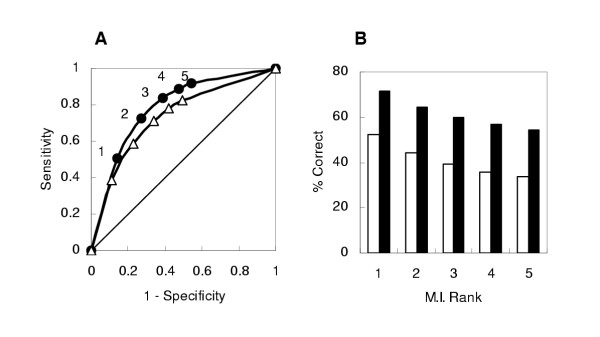
**Assigning GO term to motif according to rank of M.I. A**. ROC curves of assigning GO terms to motifs according to rank of mutual information. The filled circle is for the perfect match data set, and the area under the curve is 0.782. The empty triangle is for the relaxed match data set, and the area under the curve is 0.735. The numbers next to data points indicate cut-off ranks of decision rules. Diagonal line corresponds to random model. **B**. Precision of rules based on different mutual information cutoff ranks. Filled bars are results on the perfect match data set. Empty bars are the results on the relaxed match data set.

Figure 2 shows the Receiver Operating Characteristic (ROC) curve [20] of assigning GO terms to PROSITE motifs in our data set according to the rank of motif-term associations. The two curves are for the two different labeled association sets (i.e., perfect match and relaxed match) respectively. The areas under the two curves are 0.782 and 0.735 respectively, which can be considered as fairly good. We also plot the precision, also referred to as positive predictive value, in panel B. The precision is calculated as the percent of predicted assignments that are truly correct. As shown in panel B, if we assign the GO terms at the top rank for all PROSITE motifs, 50–70% of the cases will be predicted correctly. As we loosen the threshold to include lower ranked terms, we would assign more terms to a motif, and as expected, precision would decline. But even at rank 5, we still have a precision of about 50%. Also shown in Table 2, with respect to the PROSITE pattern of tyrosine kinase (PS00109), most of the top five associated GO terms are related to kinase activity and the term with the highest rank is the most specific.

### Predicting motif functions using logistic regression

While the mutual information measure appears to give reasonable results, there are three motivations for exploring more sophisticated methods. First, the mutual information value is only meaningful when we compare two candidate terms for a given motif pattern; it is hard to interpret the absolute value. While a user can empirically tune the cutoff based on some utility preferences, it would be highly desirable to attach some kind of confidence value or probability of correctness to all the potential candidate motif-term associations. Second, there may be other features that can also help predict the function (term) for a motif. We hope that the additional features may help a classifier to further separate correct motif-term assignment from wrong ones. Third, there exist many motifs with known functions (e.g., those in the PROSITE database), and it is desirable to take advantage of such information to help predict the functions of unknown motifs. This means that we need methods that can learn from such information. In this section, we show that the use of logistic regression can help achieve all three goals. Specifically, we use logistic regression to combine the mutual information with other features, and produce a probability of correct assignment. The motifs with known functions serve as training examples that are needed for estimating the parameters of the regression function.

### Feature extraction and parameter estimation

We now discuss the features to be used in logistic regression, in addition to the mutual information discussed in the previous section. The goal is to identify a set of features that is helpful to determine whether association of any pair of a GO term and a motif is correct or not, without requiring specific information regarding the function of GO term and motif. For a distinct motif-term pair, we collect following frequency-based features: (1) The number of sequences in which the GO term (*T*) and PROSITE motif (*M*) co-occur (*NT-M*). (2) The number of sequences in which *T *occurs (*NT*). (3) The number of sequences in which M occurs (*NM*). (4) The number of distinct GO terms (*G*) seen associated with *M *(*NG|M*). (5) The number of distinct PROSITE patterns (*P*) seen associated with *T *(*NP|T*). In addition, we also consider, as a feature, the similarity of the sequences that support a motif-term pair. Intuitively, if a motif is conserved among a set of diverse sequences, it is more likely that the motif is used as a building block in proteins with different functions. Thus, the average pair-wise sequence similarity of the sequence set can potentially be used as a heuristic feature in the logistic regression classifier. Given a set of sequences, we use a BLAST search engine to perform pair-wise sequence comparisons. We devised a metric *AvgS *to measure the averaged pair-wise sequence similarity per 100 amino acids (see methods) and use it as an input feature for classifier.

To cast the prediction problem as a binary classification problem, we augment our data set of motif-term pairs with a class label variable *Y*, so that *Y *= 1 means correct assignment and 0 means incorrect. We represent a motif-term pair by a vector of features ***X ***= (*X*_1_,..., *X*_*k*_), where *k *is the number of features. The seven features/variables used in our experiments are *NT-M, NT, NM, NG|M, NP|T, AvgS*, and *M.I.*. Suppose we have observed *n *motif-term pairs, then we have *n *samples of (*y*_*i*_, ***x***_*i*_), *i *= 1, 2, ..., *n*, where, *y*_*i *_is the correctness label and ***x***_*i *_is the feature vector for the corresponding motif-term pair. Our goal is to train a classifier which, when given a motif-term pair and feature vector ***X***, would output a label *Y *with value 1 or 0. Alternatively, we can also consider building a classifier which outputs a probability that *Y *= 1 instead of a deterministic label. Thus, our task is now precisely a typical supervised learning problem; many supervised learning techniques can potentially be applied. Here, we choose to use logistic regression as our classification model because it has a sound statistical foundation, gives us a probability of correct assignment, and can combine our features naturally without any further transformation.

**Figure 3:**
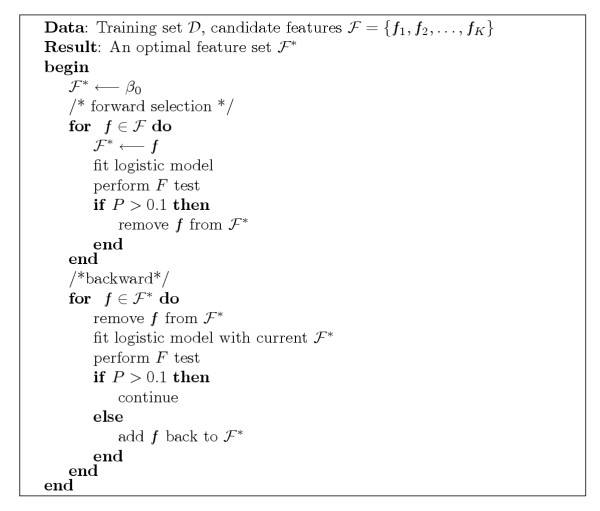
The algorithm for feature selection.

In order to build a model only with the truly discriminative features, it is a common practice to perform feature selection for logistic regression. We use a combined forward and backward feature selection algorithm. Starting from the intercept, we sequentially add features into the model and test if the log-likelihood increases significantly; we keep the current feature if it does. After the forward selection, we sequentially drop features from the model, to see if dropping a feature would significantly reduce the log-likelihood of the model; if it does, we exclude the feature from the model, otherwise continue. When testing the significance, we use the likelihood ratio statistic *G*, given by 2*l*(*D|β *_*f*_)/*l*(*D|β *_-*f*_), where, *l*(*D|β *_*f*_) and *l*(*D|β *_-*f*_) are the log-likelihood of the model with feature *f *and the model without feature *f*, respectively. Since we add or drop one feature at a time, *G *follows χ^2 ^distribution with degree of freedom of 1 [21]. We use the *p*-value of 0.1 as a significant threshold. Figure 3 illustrates the procedure of feature selection. We found that the average pair-wise similarity of supporting sequence set does not contribute to the model significantly and so excluded it; all other variables contribute to the model significantly. The results of parameters estimation are show in the Table 3.

### Logistic regression classification

After fitting the model using the training set, we tested the model on the test set, i.e., we used the model to compute an output *p*(*Y*_*i *_= 1|***X***_*i*_) for each test case. Table 4 shows an example of computed conditional probability of correct assignment for the GO terms associated with the protein motif possible the motif "PS00383", which is the "tyrosine specific protein phosphatases signature and profiles". The table 4 lists top 5 GO terms, which are observed to be associated with the motif and ranked according to the conditional probability returned by logistic regression.

**Figure 4:**
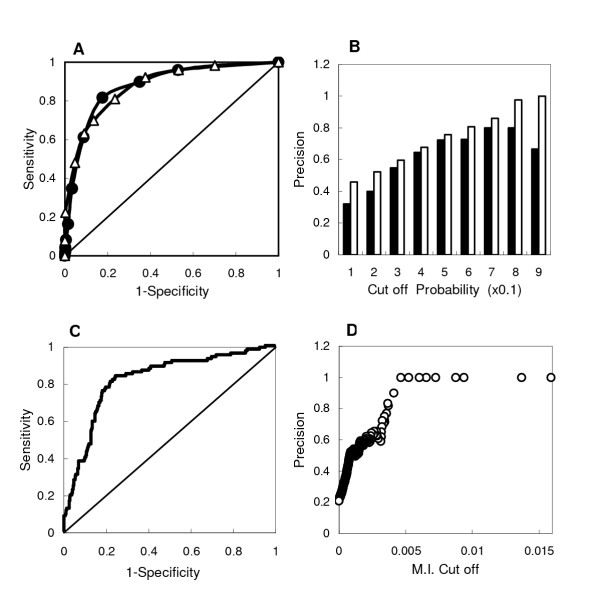
**Comparison of results with probability and M.I. A**. ROC curves for classifying motif-term associations at different probability threshold. Filled circles are the results on the perfect match test set with an area under curve of 0.8715. Empty triangles are on the relaxed match test set with an area under curve of 0.871. Data points correspond to thresholds of p(*Y *= 1|***X***) from 0.9 to 0.1 (from left to right) with a step of 0.1. **B**. Precision (positive predictive value) at different probability cutoffs, where solid bars are the result on the perfect match test set and the open bars for the relaxed match test set. **C**. ROC curve for decision rules based on different M.I. cutoff thresholds with an area under curve of 0.816. **D**. Precision at different M.I. cutoffs.

As the results from the logistic regression are the conditional probability that an association of a GO term with a given motif is correct, we need to decide the cut off threshold for making decision. We calculate the sensitivity and specificity for a different threshold from 0.1 to 0.9 with a step of 0.1 and plotted the ROC curves as shown in Figure 4. The areas under the logistic regression ROC curves are 0.875 and 0.871 for perfect match and relaxed match test set respectively. The precision of the rules is plotted in panel B, where we see that, as the rule becomes more stringent (using a higher threshold), predictions generally become more accurate. We noticed that the precision on the perfect match test set is more variable. This is probably due to the fact that this data set has fewer cases with *Y *= 1, thus, a small change in the number of cases introduces a large change in percentage. For example, when the threshold is set at 0.9, only three cases are covered by the rule and two of them are correct, thus percent correct drop to 66%.

To see whether the additional features are useful, we also performed ROC analysis using different mutual information cutoff threshold on the perfect match test set. The result is shown in Figure 4 panels **C **and **D**. We see that using mutual information alone performs almost as well as logistic regression with additional features. However, the area under the curve (0.816) is smaller than that of logistic regression (0.875), indicating that logistic regression does take advantage of other features and has more discriminative power than mutual information alone.

The coefficients *β*_1_, *β*_2 _and *β*_3 _for the three features *NT-M*, *NT *and *NM*, which are also involved in the calculation of mutual information, have a very interesting interpretation – they indicate that the roles of these three variables in the logistic regression model actually are to compromise the effect of mutual information! Indeed, according to the formula of the mutual information, a strong correlation corresponds to a high *NT-M*, low *NT*, and low *NM*, but the coefficients shown in Table 3 clearly suggest the opposite. We believe that this actually corrects one drawback of mutual information – over-emphasizing the correlation but ignoring the support or the strength of evidence. For example, if a term is rare, say occurs only once in the data set, then it would have a very high mutual information value (due to an extremely low *NT*) with respect to any pattern matched by the sequence to which the term is assigned. But, intuitively, one occurrence is very weak evidence, and at least should be regarded as weaker than when we have a term occurring 10 times in total and co-occurring 9 times with the same motif. The key issue here is that mutual information only reflects the correlation between variables, but does not take into account the strength of evidence, therefore, tends to over-favor the situation where there is a perfect correlation but very little evidence. However, the number of sequences in which the co-occurrence happens, which is called the "support" for the association, is also very important.

The coefficients for the other two parameters, *NG|M *and *NP|T*, are also meaningful. Their negative signs indicate that the more terms a motif co-occurs with or the more motifs a term co-occurs with, the less likely a particular association is correct. This also makes sense intuitively, since all those co-occurring terms can be regarded as "competing" for a candidate description of the motif's function, so the more terms a motif is associated with, the competition is stronger, and thus the chance that any particular term is a correct description of function should be smaller. Thus, the logistic regression model not only performs well in terms of prediction accuracy but also gives meaningful and logically plausible coefficient values.

## Discussion

In this paper, we explore the use of the Gene Ontology knowledge base to predict the functions of protein motifs. We find that the mutual information can be used as an important feature to capture the association between a motif and a GO term. Evaluation indicates that, even used alone, the mutual information could be useful for ranking terms for any given motif. We further use logistic regression to combine mutual information with several other statistical features and to learn a probabilistic classifier from a set of motifs with known functions. Our evaluation shows that, with the addition of new features and with the extra information provided by the motifs with known functions, logistic regression can perform better than using the mutual information alone. This is encouraging, as it shows that we can potentially learn from the motifs with known functions to better predict the functions of unknown motifs. This means that our prediction algorithm can be expected to further improve, as we accumulate more and more known motifs.

Although we have so far only tested our methods on the known motifs, which is necessary for the purpose of evaluation, the method is most useful for predicting the functions of new and unknown motifs. For the future work, we can build a motif function prediction system and apply our algorithm to many candidate *new *motifs e.g., those discovered using TEIRESIAS, SPLASH or other programs. This would further enable us to perform data mining from the Gene Ontology database in several ways. For example, we can hypothesize the functions of a large number of novel motifs probabilistically, then we will be able to answer a query, such as "finding the five patterns that are most likely associated with the GO term tyrosine kinase". This is potentially very useful because it is not uncommon that substantial knowledge about the functions and sub-cellular location of a given protein is available even though a structural explanation for the functions remains obscure. On the other hand, we believe that our methods will facilitate identifying potentially biological meaningful patterns among the millions of patterns returned by pattern searching programs. A sequence pattern that associates with certain GO term with high M.I. or probability is more like to be a meaningful pattern that that with low scores. Furthermore, our methods can also be used in automatic annotation of novel protein sequences as suggested in Schug et al and Rigoutsos et al [9,13,22]. Our methods provide different approaches to associate sequence patterns with functional descriptions. After associating functional descriptions (in the form of GO term) to motifs, we can determine what motifs a novel protein sequence matches and correspondingly transfer the functional descriptions associated with motifs to the sequence. One key advantage of our methods is that the probability of correctness for a GO-motif association can be considered as confidence or uncertainty. This enables one to optimize the automatic annotation according to Bayesian decision theory and minimize the risk of incorrect annotation.

Having stated the potential uses of our approaches, we also realize that there exist some limitations for our methods. For example, in order to predict the function of a newly identified sequence pattern correctly, we would require functional annotations of the sequences of GO database be complete and accurate, which may not always be the case. In this paper, we mainly used the motifs with known function to evaluate the capability of the methods developed in this research. Our result shows that the methods work well with known sequences patterns. Currently, the annotation of motif function with GO term is carried out manually at the European Bioinformatics Institute (the GOA project). Such approach is warranted because human annotation is more accurate than automatic ones. However, as the amount of information regarding protein functions accumulates and a large number of new potential motifs are discovered, it will be very labor intensive to annotate the potential association of protein function and protein patterns. By then, the methods studied in this research will potentially prove to be useful to discover the underlying protein motifs that are responsible for the newly annotated function. For example, the methods can be used as prescreening to narrow down to the most possible associations of protein function and motifs, thus facilitate human annotation.

## Conclusions

In summary, we have developed methods that disambiguate the associations between of Gene Ontology terms and protein motifs. These methods can be used to mine the knowledge contained in the Gene Ontology database to predict the function of novel motifs, discover the basis of a molecular function at primary sequence level and automatically annotated the function of novel proteins.

## Methods

### Mutual information

Mutual information is defined as follows


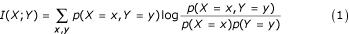


In which the probabilities *p*(*X *= *x*, *Y *= *y*), *p*(*X *= *x*) and *p*(*Y *= *y*) can be empirically estimated from the data by counting occurrence/co-occurrence followed by normalization.

### Sensitivity and specificity

The sensitivity and specificity of the rules are calculated as


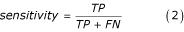



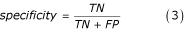


where *TP *(True Positive) is the number of associations labeled as correct among the retrieved motif-term pairs meeting the ranking cutoff criteria, *FN *(False Negative) is the number of associations labeled as correct but not retrieved, *TN *(True Negative) is the number of associations labeled as incorrect and not retrieved, and *FP *(False Positive) is the number of associations labeled incorrect but are retrieved.

### Averaged sequence similarity

Calculation of the average pair-wise sequence similarity per 100 amino acids (*AvgS*) of a sequence set is as follows


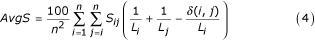


Where S_*ij *_is raw BLAST pair-wise similarity scores between the sequence *i *and sequence *j*; *L*_*i *_and *L*_*j *_are the lengths of sequences *i *and *j*, respectively; *n *is the number of sequences in the set; and *δ *(*i*, *j*) is a delta function which equals 1 if *i *= *j *and 0 otherwise.

### Logistic regression

The logistic regression model is a conditional model that assumes the following linear relationship between *p*(*Y *= 1|***X***) and *X*_1_, ..., *X*_*k*_:


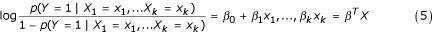


where, *β *= (*β*_0_, *β*_1_, ..., *β*_*k*_) is the parameter vector. We can fit the logistic regression model (i.e., estimate the parameters) using the Maximum Likelihood method – essentially setting the parameters to values at which the likelihood of the observed data is maximized (Hosmer and Lemeshow 1989, Hastie et al 2001). In our experiments, we use iteratively reweighted least squares (IRLS) algorithm [23] to fit the logistic regression model. All features are normalized to zero mean and unit variance before training.

## Figures and Tables

**Table 1:** GO terms and PROSITE patterns for the protein MGI|MGI:97380

**GO Terms and Descriptions**		**PROSITE Motifs and Descriptions**	
GO:0005576	Extracellular	PS00248	Nerve growth factor family signature and profile (1)
GO:0005515:	Protein-binding	PS50270	Nerve growth factor family signature and profile (1)
GO:0005166:	neurotrophin p75 receptor ligand		
GO:0008544	epidermal differentiation		
GO:0007422	peripheral nervous system development		
GO:0007420	brain development		
GO:0007403	determination of glial fate		

**Table 2:** Five GO terms associated with PROSITE pattern PS00109 (tyrosine kinase signature)

GO Term	GO Definition	NM	NT	NT-M	M.I.
GO:0004713	Protein tyrosine kinase	246	68	51	0.00599
GO:0006468	Protein amino acid phosphorylation	246	409	69	0.00464
GO:0004714	Transmembrane receptor protein kinase	246	33	29	0.00362
GO:0004715	Non-transmembrane protein tyrosine kinase	246	17	14	0.00168
GO:0005887	Integral membrane protein	246	1162	44	0.00118

**Table 3:** Result of logistic regression parameters estimation

	Estimated Coefficients	
	
Features	On the perfect match set	On the relaxed match set
Intercept (*β*_0_)	-1.7549	-0.6263
NT-M(*β*_1_)	-0.3845	-0.4546
T (*β*_2_)	1.3652	1.6827
NM (*β*_3_)	1.0497	0.4735
NG|M (*β*_4_)	-1.9792	-1.1113
NP|T (*β*_5_)	-1.7883	-2.5494
M.I. (*β*_6_)	1.0002	1.1598

**Table 4:** Top 5 GO terms associated with the motif PS000383 ranked according the conditional probability of correctness of association. The column 2~7 consist of the feature vector for motif-GO association is listed, the conditional probability p(*Y *= 1|***X***) is calculated with trained model and the true classes are list in right two columns of the table. The definition of the GO terms is listed at the bottom of the table.

GO Terms	Input Features (***X***)						p(*Y *= 1|***X***)	True Class
	NT-M	NT	NM	NG|M	NP|T	M.I		
			
GO:0006470	65	122	191	95	27	0.007268	0.97577	1
GO:0005001	23	24	191	95	5	0.003177	0.87681	1
GO:0004726	10	11	191	95	10	0.001331	0.68682	1
GO:0005634	11	1785	191	95	281	5.08E-06	0.27882	0
GO:0005887	17	1162	191	95	225	0.000193	0.15536	0

GO:0006470: Protein amino acid dephosphorylation; Cellular Processes

GO:0005001: transmembrane receptor protein tyrosine phosphatase activity: Molecular Function

GO:0004726: non-membrane spanning protein tyrosine phosphatase activity: Molecular Function

GO:0005634: Nucleus; Cellular Component

GO:0005887: Integral to plasma memberane; Cellular Component
